# The effects of exercise on vascular endothelial function in type 2 diabetes: a systematic review and meta-analysis

**DOI:** 10.1186/s13098-018-0316-7

**Published:** 2018-03-06

**Authors:** Jung-Hoon Lee, Ruda Lee, Moon-Hyon Hwang, Marc T. Hamilton, Yoonjung Park

**Affiliations:** 1 Laboratory of Human Physiology, Korea National Sport University, Seoul, Republic of Korea; 2 Exercise & Cardiovascular Physiology Laboratory, Division of Health and Exercise Science, Incheon National University, Incheon, Republic of Korea; 3 Sport Science Institute, Incheon National University, Incheon, Republic of Korea; 4 Texas Obesity Research Center from the Division of Research, and Department of Health and Human Performance, University of Houston, Houston, TX USA; 5 Laboratory of Integrated Physiology, Department of Health and Human Performance, University of Houston, 3875 Holman St, Houston, TX 77204-6015 USA

**Keywords:** Type 2 diabetes, Exercise training, Endothelial function, Flow mediated dilation, NO bioavailability, Sedentary, Low intensity exercise

## Abstract

**Background:**

Vascular endothelial dysfunction induced by hyperglycemia and elevated insulin resistance is a potent risk factor for cardiovascular disease and likely contributes to multiple chronic disease complications associated with aging. The aim of this study was to systematically review and quantify the effects of exercise on endothelial function (EF) in type 2 diabetes (T2D).

**Methods:**

Five electronic databases were searched (until June 2017) for studies that met the following criteria: (i) randomized controlled trials; (ii) T2D aged ≥ 18 years; (iii) measured EF by brachial artery flow-mediated dilation (FMD); (iv) structured and supervised exercise intervention for ≥ 8 weeks.

**Results:**

Thirteen cohorts, selected from eight studies (306 patients, average age 59 years), met the inclusion criteria. Exercise training significantly increased FMD (mean ES = 0.41, 95% CI 0.21–0.62, *P* < 0.001). Low to moderate intensity subgroups and aerobic exercise (AE) subgroups significantly increased FMD more than moderate to high intensity subgroups and combined AE and resistance exercise subgroups respectively (*P* < 0.01, *P* < 0.05). The Grading of Recommendations Assessment, Development and Evaluation (GRADE) assessments reported that quality of evidence for all outcomes was moderate except shear rate showing low. Egger’s test showed no significant publication bias for all outcomes.

**Conclusion:**

Our results suggest that in patients with T2D, lower intensity exercise has physiological meaningful effects on EF, in support of the emerging concept that the lower efforts of exercise are not necessarily less cardioprotective than higher intensity training.

## Background

Type 2 diabetes (T2D) is one of the major risk factors for cardiovascular disease (CVD) [1]. According to American Heart Association, CVD in older adults with T2D accounts for 84% of the deaths [2]. Vascular endothelial dysfunction is related to elevated blood glucose level and insulin resistance and is a major cause in the pathological progression towards CVD [3, 4]. Endothelial dysfunction is considered a precursor of atherosclerosis and CVD [5] because the vascular endothelium plays an important physiological role in vascular homeostasis [6]. In most clinical and physiological settings, the direct physiological or biochemical effects signaling within the endothelium are more associated with endothelial dysfunction than smooth muscle dysfunction per se [7]. Vascular endothelial and smooth muscle cells release and respond to the internally generated substances including nitric oxide (NO) to regulate the vascular relaxation and tone [8].

Regular physical activity has been recommended as an effective treatment together with medications and dietary control to improve vascular endothelial function (EF) in T2D. Skeletal muscle contraction during physical activity increases local blood flow and cardiac output, which results in increased shear stress on vascular endothelium and increased NO production [9]. From a review of six previous studies, Way et al. [10] concluded that exercise training did not result in a significant effect on EF in T2D , whereas Montero et al. [11] observed that exercise training in four studies improved EF in T2D. There were mixed results, potentially in part, because of the small number of participants in which EF was evaluated by brachial artery FMD, the gold standard measure of EF [12].

Therefore, the primary purpose of this study was to evaluate the effects of exercise training on EF measured by brachial artery FMD in adults with T2D by conducting a systematic review and meta-analysis. In doing this, we carefully examined the specifics of the exercise training regimens (such as intensity, modality, duration, and frequency of exercise) and the reported body mass index (BMI) in order to provide more evidence for designing exercise programs for T2D patients at risk of CVD.

## Methods

This current systematic review followed the strategy of The PRISMA Statement [13].

### Data sources

Five electronic databases (CINAHL, EMBASE, PubMed, SportDiscus, and Web of Science) were searched for eligible studies published in English from the earliest date available to June 2017. The following keywords were used for searches: ‘exercise or training or physical activity’, ‘flow mediated dilation’, and ‘type 2 diabetes’. Manual searches of reference lists were conducted to ensure all relevant studies were captured. Two reviewers (Lee JH and Lee RD) independently searched all of the articles and applied the inclusion and exclusion criteria to the titles and abstracts searched. Disagreements about the inclusion and exclusion were resolved by another reviewer (MH, Hwang). When the information was not clear, the full text papers of the studies were obtained for review. Corresponding authors of potentially eligible studies were contacted if studies reported data for which it was impossible to discriminate.

### Study selection

The inclusion criteria for eligible studies were as follows: (i) randomized controlled trials; (ii) adult humans aged ≥ 18 years who have T2D which was defined by the World Health Organization and the American Diabetes Association’s criterion of fasting plasma glucose ≥ 7.0 mmol/l or 126 mg/dl; (iii) studies that measured EF by brachial artery FMD; (iv) structured and supervised exercise intervention for ≥ 8 weeks. Studies were excluded if T2D patients have a neurological complication, diabetic neuropathy. Duplicate studies or sub-studies of included trials were also excluded from the analysis. Trials including dietary supplements or caloric restriction were excluded to focus on the effects of exercise alone.

### Quality assessment

Two reviewers (Lee JH and Lee RD) independently assessed the quality of the included studies using the PRISMA recommendations [13]. The quality assessment consisted of six items: (i) appropriate generation of random allocation sequence; (ii) concealment of the allocation sequence; (iii) blinding of the assessment and collection outcomes; (iv) proportion of participants lost to follow-up; (v) complete outcome data; (vi) the intention-to-treat principle [13]. Where reviewers disagreed, specific criteria were discussed with a third reviewer (Hwang MH) until consensus was reached. In addition, the overall quality of the evidence was assessed using the Grading of Recommendations Assessment, Development and Evaluation (GRADE) [14]. Based on this assessment, the intervention was graded accordingly: ‘high quality’—we are very confident that the true effect lies close to that of the estimate of the effect; ‘moderate quality’—we are moderately confident in the effect estimate. The true effect is likely to be close to the estimate of the effect, but there is a possibility that it is substantially different; ‘low quality’—our confidence in the effect estimate is limited: the true effect may be substantially different from the estimate of the effect; ‘very low quality’—we have very little confidence in the effect estimate: the true effect is likely to be substantially different from the estimate of the effect [15].

### Data extraction

Data were extracted from all selected studies by two independent reviewers to record the detailed information in terms of subject characteristics, study methods, interventions, outcomes, and adverse events. We used means and standard deviation (SD), but where standard errors or 95% confidence interval (CI) were provided, they were converted to SD. Corresponding authors were contacted for detailed information where required.

In terms of population characteristics, age, gender, BMI, number of participants, complications, and duration of T2D of participants were recorded to compare the similarity of participants between trials. The primary outcomes were FMD including shear rate and baseline diameter, and secondary outcome was BMI. Brachial artery was only selected instead of femoral or popliteal artery for the measurement of FMD because it was more possible to compare across multiple studies from the brachial arm measurements and the assessment of brachial EF plays a role in predicting CVD and atherosclerosis [16]. Regarding intervention, we recorded total duration, frequency (days per week), intensity, session duration, type and order of exercise, names of exercise machine or tool, supervisors, and places of intervention to compare the similarity of training methods between trials. The median values were used for calculation if the studies reported a range of data (e.g. 16, 15–17 of repetitions). Detailed interventions about control groups (CON) and any additional supplements were recorded. Measurement technique and region were also extracted.

### Data analysis

Heterogeneity between studies was assessed using the Cochran *Q* statistic [17] and the *I*^2^ test [18]. *I*^2^ ranges from 0 to 100%: a value < 25% indicates low risk of heterogeneity, 25–75% indicates moderate risk of heterogeneity, and > 75% indicates high risk of heterogeneity. In each study, the effect size (*ES*) for the intervention was calculated by the difference between the means of the post-measurement and pre-measurement at the end of the intervention using Hedges g. Separate meta-analyses of trials with FMD, shear rate, baseline diameter, and BMI were performed to generate the mean *ES* and 95% CI. *ES*s were classified according to Cohen’s definition (1988), where 0.2 is considered small, 0.5 moderate, and 0.8 large [19]. We used a fixed-effects model when homogeneity was verified or a random-effects model when heterogeneity was shown by the *Q* statistic [18]. Where multiple intervention groups were included in one study, we split the shared group into two or more groups with smaller sample size [20]. Publication bias was assessed using the Egger’s regression test [21]. To evaluate whether an individual cohort had undue influence on the overall meta-analysis result, we performed sensitivity analyses in all four outcomes by omitting one of the trials at a time and determining whether statistical conclusion remained the same. All calculations were conducted with SPSS version 20, Microsoft Excel 2016, and STATA version 14.2.

Subgroup analyses were performed where sufficient numbers of trials existed in subgroups to identify potential factors influencing the effect of exercise on outcomes and accounting for the heterogeneity between studies: (i) age < 60 versus age ≥ 60; (ii) low baseline BMI levels (< 30 kg/m^2^) versus high baseline BMI levels (≥ 30 kg/m^2^); (iii) low baseline glycated hemoglobin (HbA1c) levels (6.5–7.5%) versus high baseline HbA1c levels (> 7.5%); (iv) low baseline FMD levels (≤ 4.8%) versus high baseline FMD levels (> 4.8%); (v) AE versus combined AE and RE; (vi) low to moderate intensity versus moderate to high intensity; (vii) 8 weeks versus 12 weeks or more; (viii) less than 60-min versus 60-min or more. Random effects meta-analysis regression was conducted to compare the effect estimates (effect size) in different subgroups by considering the meta-analysis results from each subgroup separately. To interpret the results of subgroup analyses, *P* value (*P* < 0.05) between study variation was considered for the statistical difference between subgroups.

## Results

### Study selection and characteristics

**Fig. 1:**
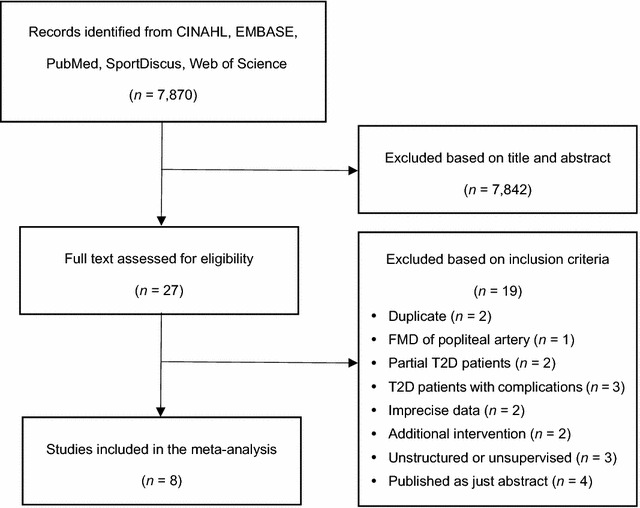
Study search and selection process. *FMD* flow-mediated dilation, *T2D* type 2 diabetes

The search resulted in 7870 potential studies (Fig. 1). From the titles and abstracts, 7842 studies were excluded based on the criteria, and then 27 full text studies were reviewed. Of these, 19 articles were excluded; two articles were duplicate [22, 23], one measured FMD of popliteal artery [24], two had patients with different kinds of diseases as well as T2D in the groups [25, 26], three had T2D patients with peripheral arterial disease or diabetic peripheral neuropathy [27–29], two did not provide precise data [30, 31], two additionally treated dietary control for weight loss [32, 33], three involved unstructured or unsupervised exercise intervention [34–36], and four were just abstracts with incomplete data [37–40]. Four exercise groups (EX) with additional interventions were included after discussing because there was no difference in the effect of interventions on FMD, our major outcome, between the CON and experimental groups: (i) exercise in the hypoxic environment (16.5% O_2_, 2000 m) [41]; (ii) endothelin (ET) receptor blockade or a placebo [42]; (iii) walking meditation [43]. One AE trial which recorded the time, frequency, and intensity of exercise by a multi-record accelerometer were also included because the subjects visited the laboratory every 1 or 2 weeks [44]. Moreover, two CON including AE on treadmill [43] and combined AE and RE in the normoxic environment [41] were included in the EX. Eventually, we selected thirteen exercise trials in eight studies by consensus.

### Participants

Table 1 shows the characteristics of all of the studies included. Articles were published from January 2010 [45] to June 2016 [43]. The sample size was 316. Of these, 10 healthy participants in the CON [46] were excluded for our analysis. 306 participants completed their intervention (EX: 196, CON: 110, female%: 45%) ranging from 18 [42] to 112 [47] participants. The average age of the participants was 59 years (EX: 59 ± 7.2, CON: 58 ± 6.4). Other results from baseline measures were as follows: (i) BMI: 30.3 (EX: 30.0 ± 4.4, CON: 30.7 ± 3.6); (ii) HbA1c: 7.2% (EX: 7.4 ± 1.4, CON: 7.1 ± 1.2%); (iii) FMD: 5.5% (EX: 5.1 ± 3.5, CON: 5.9 ± 4.0).

**Table 1:** Summary of included studies

Study	Age		Number of subjects		BMI (kg/m^2^)		Baseline FMD (%)		Intervention
	EXP	CON	EXP	CON	EXP	CON	EXP	CON	EXP
Gainey et al. [43]	58 ± 10.4	63 ± 6.6	2/10	2/9	27.1 ± 4.8	26.6 ± 4.6	5.6 ± 4.2	3.9 ± 3.0	AE with walking meditation, 12 weeks, 3/week, 50 m, 50–70% HRmax
Gibbs et al. [47]	58 ± 5	56 ± 6	32/17	37/26	32.3 ± 5.3	33.5 ± 4.3	6.0 ± 4.0	6.2 ± 4.3	Combined AE and RE, 26 weeks, 3/week, > 60 m, 60–90% HRmax, 50% 1RM, 7 × 2 × 12–15
Kwon et al. [44]	AE:55.5 ± 8.6 RE:56.3 ± 6.1	58.9 ± 5.7	0/13 0/12	0/15	26.7 ± 2.6 27.4 ± 2.1	27.0 ± 2.3	4.3 ± 1.6 4.9 ± 2.5	4.7 ± 1.9	AE vs RE, 12 weeks, AE: 5/week, RE: 3/week, 60 m, AE: 3.6–6.0 METs, RE: 40–50% 1RM, 10 × 3 × Not specified
Mitranun et al. [48]	CONT: 61.7 ± 10.1 INT:61.2 ± 10.5	60.9 ± 9.3	5/9 5/9	5/10	29.4 ± 2.6 29.6 ± 1.9	29.7 ± 1.5	4.8 ± 6.0 5.4 ± 4.1	5.1 ± 5.0	CONT vs INT AE, 12 weeks, 3/week, 30–40 m, CONT: 60–65% VO2peak, INT: 80–85% (1 m) and 50–60% VO2peak (4 m)
Okada et al. [45]	61.9 ± 8.6	64.5 ± 5.9	10/11	11/6	25.7 ± 3.2	24.5 ± 2.9	7.3 ± 4.7	6.4 ± 3.6	Combined AE and RE, 12 weeks, 3–5/week, 65 m, AE: 60% HRmax, RE: N/A
Schreuder et al. [41]	57 ± 6	52 ± 8	9/1	5/4	30.9 ± 4.1	36.0 ± 6.5	3.6 ± 2.0	4.7 ± 0.6	Combined AE and RE, 8 weeks, 3/week, > 60 m, 70–75% HRR, RE: N/A, in hypoxia (16.5% O_2_)
Schreuder et al. [42]	60 ± 5	59 ± 6	8/0	10/0	32.8 ± 8.3	31.9 ± 4.6	4.3 ± 1.2	3.6 ± 2.3	Combined AE and RE (Circuit), 8 weeks, 3/week, 60 m, 70–75% HRR, 12RM, 6 × 3 × 12, ET receptor blockade
Schreuder et al. [46]	59 ± 6	58 ± 7	13/0	10/0	32.4 ± 4.2	26.9 ± 3.5	3.4 ± 2.1	3.9 ± 1.9	Combined AE and RE (Circuit), 8 weeks, 3/week, 60 m, 70–75% HRR, 12RM, 6 × 3 × 12

Values are mean ± SD

*EXP* experimental group, *CON* control group, *BMI* body mass index, *FMD* flow-mediated dilation, *AE* aerobic exercise, *RE* resistance exercise, *CONT* continuous training, *INT* interval training, *wk* week, *a/wk* days per week, *m* minutes, *HRmax* maximum heart rate, *1RM* one-repetition maximum, *METs* metabolic equivalents, *VO2peak* peak oxygen consumption, *HRR* hear rate reserve, *A* *×* *B* *×* *C* number of exercise × sets × repetitions, *ET* endothelin

### Interventions

All interventions, except for one AE group [44] using a multi-record accelerometer, were supervised in research centers by a fitness coach, researchers, or physiotherapists. The mean training period was 12 weeks (minimum–maximum: 8 [41, 42, 46] to 26 [47] weeks). Mean session duration was 57-min (minimum–maximum: 30 [48] to 80 [41, 47] min). In most studies, training frequency was 3 days per week (with the exception of [45] and [44] where 3–5 and 5 days per week were completed respectively). Of thirteen trials, five conducted AE [43, 44, 48], seven conducted a combination of AE and RE [41, 42, 45–47], and one conducted RE [44].

AE trials expressed intensity as a percent of maximum heart rate (HRmax), hear rate reserve (HRR), peak oxygen consumption (VO_2peak_) or metabolic equivalents (METs). Intensities ranged from low to moderate (50–70% of HRmax [43, 45], 60–65% of VO_2peak_ [48], and 3.6–6.0 METs [44]) to moderate to high (60–90% of HRmax [47], 70–75% of HRR [41, 42, 46]). One interval training (INT) group of [48] performed at 80–85% of VO_2peak_ for 1-min with 50–60% VO_2peak_ for 4-min, which was included in the subgroup of moderate to high intensity. AE trials of [42, 46, 48] increased their intensity over the duration of the intervention, but [41] maintained their intensity and [44–47] were not known.

RE trials established their intensity by a percent of one-repetition maximum (1-RM) or 12-RM (a level which enables the participant to complete 12 repetitions). Intensities ranged from low (40–50% of 1-RM [44, 47]) to moderate (12-RM [42, 46]). Of these, four trials increased their intensity over the duration of the intervention [42, 44, 46] except for [47]. All RE trials were performed both on upper and lower body by using 6 [42, 46], 7 [47], or 10–15 [44] exercises. In [41, 45], we could not get detailed information about training, although we contacted two of the corresponding authors. Of seven combination of AE and RE, [41, 45, 47] conducted AE and RE separately and [42, 46] combined them as a circuit training. In the current study, the CON consisted of non-exercise [44, 45, 47, 48], exercise with a placebo [42], AE on treadmill [43], combined AE and RE in the normoxic environment [41], and exercise of healthy adults [46]. Of these, one healthy group [46] were excluded for our study and three remaining exercise trials [41–43] were included in the EX.

### Measurements

All thirteen trials of eight studies measured FMD to assess brachial artery EF [41–48]. Five trials measured shear rate the area under the curve (AUC) [41, 42, 46], and [47] measured peak shear rate and [48] did shear rate at rest, which were excluded for our meta-analysis. Eleven trials measured baseline diameter [41–43, 45–48]. All trials measured FMD, shear rate, and baseline diameter of the brachial artery [41–48]. There were quite differences of the inflation pressure for measuring FMD between studies: 50 mmHg above systolic blood pressure [43, 48], 200 mmHg [47], 220 mmHg [42, 45, 46], 250 mmHg [44], not known [41]. All duration of cuffing was 5-min. All trials measured BMI [41–48] but [44] was excluded for our meta-analysis because the study only provided the data at baseline.

### Effect of exercise training

#### Flow mediated dilation

**Fig. 2:**
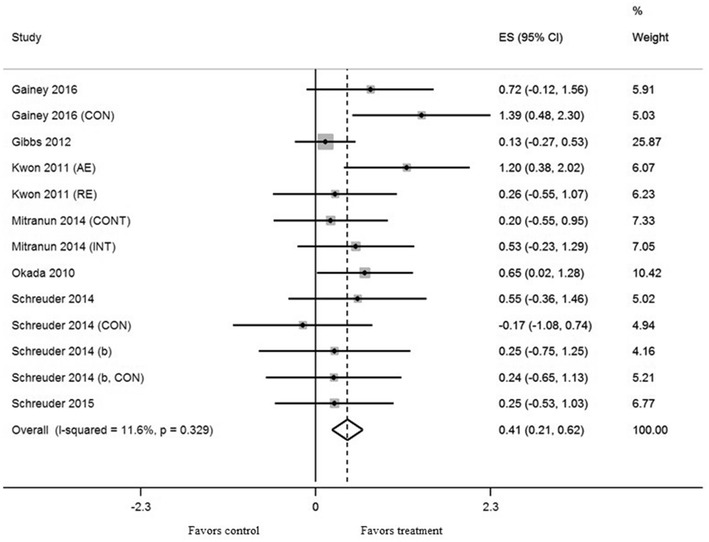
Forest plot of effect sizes 95% confidence intervals for all 13 cohorts (8 studies) representing brachial artery flow mediated dilation, based on the fixed effects results. *CON* control group, *AE* aerobic exercise, *RE* resistance exercise, *CONT* continuous training, *INT* interval training

Exercise training in thirteen trials significantly increased brachial artery FMD (mean ES = 0.41, 95% CI 0.21–0.62, *P* < 0.001) (Fig. 2). The absolute increase of FMD was 1.7%. Univariate meta-regression did not show heterogeneity between studies (*Q* = 13.57, df = 12, *P* = 0.33, *I*^2^ = 11.6%). In subgroup analysis, subgroups with low to moderate intensity significantly increased FMD more than subgroups with moderate to high intensity after training (*P* < 0.01). AE subgroups significantly increased FMD more than combined AE and RE subgroups (*P* < 0.05). There was no significant difference in effect between training for 8 weeks and ≥ 12 weeks (*P* = 0.25). Subgroups with low baseline BMI levels significantly increased FMD more than subgroups with high baseline BMI levels after training (*P* < 0.05). There was no significant difference in effect between subgroups with age < 60 and ≥ 60 (*P* = 0.28), low baseline HbA1c levels and high levels (*P* = 0.25), and low baseline FMD levels and high levels (*P* = 0.68). We excluded frequency, session duration, and other variables for the multivariate analysis because equal classifying into subgroups was impossible.

#### Shear rate and baseline diameter

**Fig. 3:**
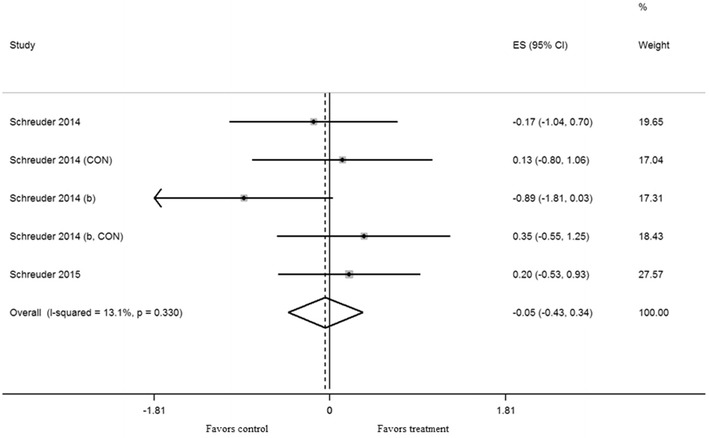
Forest plot of effect size and 95% confidence intervals for all 5 cohorts (3 studies) representing shear rate area under the curve, based on the fixed effect meta analysis result. *CON* control group

**Fig. 4:**
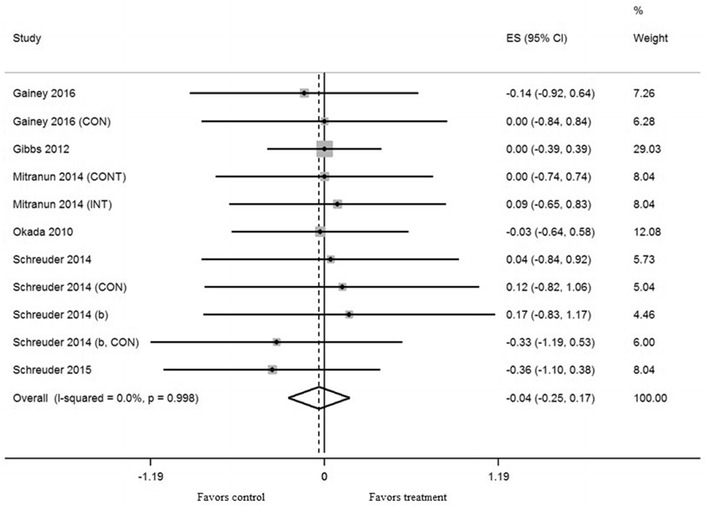
Forest plot effect size and 95% confidence intervals for all 11 cohorts (7 studies) representing baseline brachial artery diameter, based on the fixed effects meta-analysis result. *CON* control group, *CONT* continuous training, *INT* interval training

Exercise training in five trials did not result in a significant effect on shear rate AUC (mean ES = − 0.05, 95% CI − 0.43 to 0.34, *P *= 0.82) (Fig. 3). Exercise training in eleven trials did not result in a significant effect on baseline diameter (mean ES = − 0.04, 95% CI − 0.25 to 0.17, *P *= 0.72) (Fig. 4). Univariate meta-regression did not show heterogeneity between studies (shear rate, *Q* = 4.61, df = 4, *P* = 0.33, *I*^2^ = 13.1%) and (baseline diameter, *Q* = 1.69, df = 10, *P* = 0.998, *I*^2^ = 0%). Thus, we did not perform multivariate analysis with other variables.

#### Body mass index

**Fig. 5:**
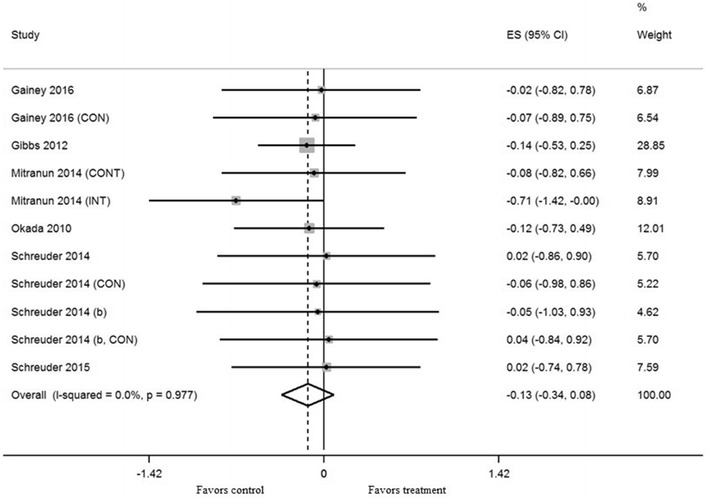
Forest plot effect size and 95% confidence intervals for all 11 cohorts (7 studies) representing body mass index, based on the fixed effects meta-analysis results. *CON* control group, *CONT* continuous training, *INT* interval training

Exercise training in eleven trials did not result in a significant effect on BMI (mean ES = − 0.13, 95% CI − 0.34 to 0.08, *P *= 0.22) (Fig. 5). Univariate meta-regression did not show heterogeneity between studies (*Q* = 3.15, df = 10, *P* = 0.98, *I*^2^ = 0%). Thus, we did not perform multivariate analysis with other variables.

#### Quality assessment and potential bias

**Fig. 6:**
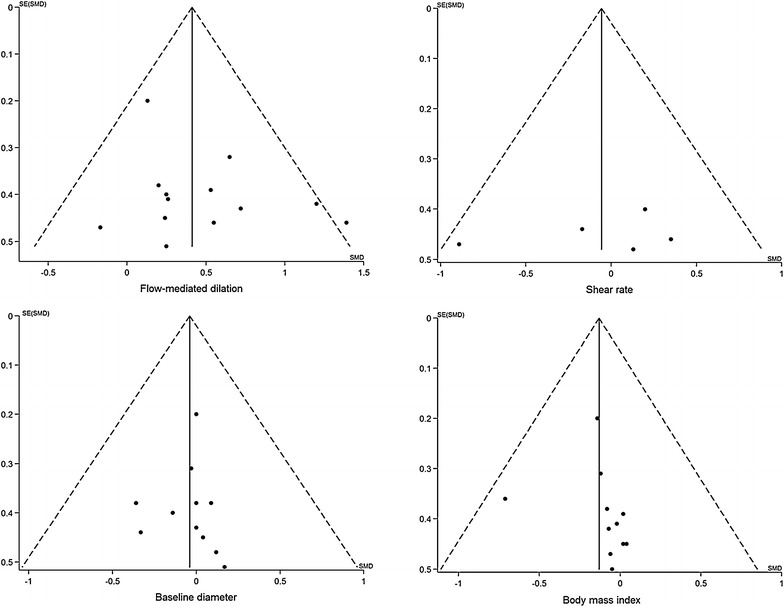
Funnel plots of publication bias in all 4 outcomes. *SE* standard error, *SMD* standardized mean difference

In the quality assessment, 88% reported appropriate generation of a random allocation sequence (7 of 8), 13% presented concealment of the allocation sequence (1 of 8), 13% described blinding of the assessment and collection outcomes (1 of 8), 100% explained proportion of participants lost to follow-up (8 of 8), 100% exhibited complete outcome data (8 of 8), and 25% reported that the intention-to-treat principle was used for statistical analyses (2 of 8). The GRADE assessments are presented in Table 2, and quality of evidence for all outcomes was moderate except shear rate AUC showing low. Egger’s test showed no significant publication bias for FMD, shear rate, baseline diameter, and BMI (*P* = 0.21, *P* = 0.58, *P* = 0.86, and *P* = 0.55, respectively) (Fig. 6).

**Table 2:** GRADE quality assessment

Quality assessment							No of patients		Effect		Quality	Importance
No of studies	Design	Risk of bias	Inconsistency	Indirectness	Imprecision	Other considerations	Exercise	Control	Relative (95% CI)	Absolute		
Flow-mediated dilation												
8	Randomised trials	No serious risk of bias	Serious^a^	No serious indirectness	No serious imprecision	None	196	110	–	SMD 0.41 higher (0.21 higher to 0.62 higher)	⊕⊕⊕Ο Moderate	Critical
Shear rate area under the curve												
3	Randomised trials	No serious risk of bias	Serious^a^	No serious indirectness	Very serious^b,c^	None	78	15	–	SMD 0.05 lower (0.43 lower to 0.34 higher)	⊕⊕ΟΟ Low	Critical
Baseline brachial artery diameter												
7	Randomised trials	No serious risk of bias	No serious inconsistency	No serious indirectness	Serious^c^	None	171	95	–	SMD 0.04 lower (0.25 lower to 0.17 higher)	⊕⊕⊕Ο Moderate	Critical
Body mass index												
7	Randomised trials	No serious risk of bias	No serious inconsistency	No serious indirectness	Serious^c^	None	183	95	–	SMD 0.13 lower (0.34 lower to 0.08 higher)	⊕⊕⊕Ο Moderate	Important

^a^Quite different inflation pressure between studies

^b^Outcome based on only three studies

^c^95% confidence interval includes zero

#### Sensitivity analysis

Sensitivity analysis reported that by excluding any of all cohorts from the meta-analysis the estimated effects will still be within the 95% CI of the mean ES in all four outcomes, suggesting the results of the meta-analysis will not significantly change after the removal of any one cohort. In terms of FMD, we conducted further sensitivity analysis by excluding two cohorts together, CON of [43] and AE group of [44] and the result did not yield a significantly different conclusion from the overall meta-analysis result (mean ES = 0.31, 95% CI 0.09–0.52, *P* < 0.01).

#### Adverse events

The presence or absence of adverse events was recorded in two of the nine studies. Two reported there were no adverse events [42, 45].

## Discussion

The primary results of this meta-analysis study are that exercise training significantly increased brachial artery FMD by 1.7% in T2D patients with an average age of 59, but there was no change in shear rate AUC, baseline diameter, and BMI.

Regular exercise-induced improvement in vascular EF measured by FMD can be attributed in large part to the increase in endothelium-derived NO production and bioavailability [49]. NO is a major vasodilator, and plays an additional role in inhibiting atherosclerotic inflammatory process, oxidative stress [49], and smooth muscle cell proliferation [50]. In the current study, however, shear rate AUC remains unchanged after training, suggesting that medium- to long-term exercise training does not necessarily require altering only this physical stimulation for triggering FMD. Exercise also enhances antioxidant capacity by increased expression of the antioxidant enzymes and reduced nicotinamide adenine dinucleotide oxidase activity [51], resulting in the increase in NO bioavailability [52]. In addition to enhanced endothelial function, the improvement in vascular smooth muscle sensitivity to NO might play a partial role in increasing FMD through promoting vasodilation. T2D patients compared to healthy population may have lowered sensitivity to NO in vascular smooth muscle, and so pathways for vasodilation were degraded [53] because elevated blood glucose can decrease a response of smooth muscle cells by increasing oxidative stress [54]. However, previous human studies generally reported that exercise training does not result in effects on vascular smooth muscle sensitivity to NO [55–57], and these results may be due to that almost all human studies used a single dose of vasodilator nitroglycerine for assessing vascular smooth muscle function without considering the effects of dose–response changes [58] and that smooth muscle adaptation to exercise training might not be observed in vivo research [59]. Further studies using the elaborate assessment of smooth muscle function in humans is required.

Subgroups with low to moderate intensity training increased FMD more than moderate to high intensity subgroups in our study. Since shear stress is a potent factor for NO release, one might expect that moderate to high intensity training would have caused greater change in FMD than low to moderate intensity due to a greater rise in cardiac output and peripheral blood flow. This unexpected result may also suggest that increased NO production and availability in T2D patients do not solely depend on an increase in flow and/or shear stress. On the other hand, shear rate is determined by diameter because shear rate is calculated as blood flow velocity divided by diameter according to Pyke and Tschakovsky [60]. In our study, baseline brachial artery diameter remained unchanged after intervention, which may explain the reason why the amount of shear rate AUC change was insignificant even though blood flow velocity increased by exercise training. Therefore, there might have been a negligible difference in shear stress between low to moderate and moderate to high intensity training because variations of shear rate might be abated by unchanged diameter although high intensity training increased more blood flow velocity than low intensity training.

However, there is certainly a growing appreciation that the optimal exercise training program for many outcomes does not necessarily follow a dose–response relationship around the relative effort, and sometimes “more is not better” as once thought. There are also other factors to consider in the present set of studies measuring FMD in patients with diabetes. Firstly, the results we found may be due to that most subgroups with low to moderate intensity performed AE whereas most subgroups with moderate to high intensity did combination of AE and RE. Although there are few studies investigating effects of RE on EF comparing with AE in T2D patients, AE is more likely to improve EF than RE or combination AE and RE. Kwon et al. reported that AE significantly increased FMD in T2D patients but RE group also showed a tendency increasing FMD [44]. In the meta-analysis study by Ashor et al., FMD in adults was increased more by AE than RE or combination of AE and RE, but the two latter groups also increased FMD significantly [61]. RE has been considered increasing vascular stiffness because RE can induce endothelin-1 [62], a potent vasoconstrictor. Also, frequently elevated blood pressure during RE may alter the arterial structure or arterial load-bearing properties [63], which can attenuate the improvement in FMD by AE. However, Miyachi [63] reported, in his meta-analysis study, that high-intensity RE results in a significant increase in arterial stiffness, but combined AE and RE can prevent arteries from stiffening by high-intensity RE. Secondly, another possible description for more favorable effects of low to moderate intensity training subgroups on EF than moderate to high intensity training subgroups is that most subgroups with moderate to high intensity conducted both AE and traditional RE using a few specific skeletal muscles at once, resulting in an increase in local blood flow, in contrast to AE promoting blood circulation in whole body. However, in the current study, *P* value of the difference in effects between training with low to moderate intensity and moderate to high (*P *= 0.007) is greater than the one between AE and combination of AE and RE (*P *= 0.028), suggesting that training intensity may be more influential factor for improving vascular EF than training modality. Moreover, combination of AE and RE in our study mostly devotes more time on AE than RE. In particular, the combined exercise training subgroups have no high intensity RE (low: 40–50% of 1-RM [44, 47] to moderate: 12-RM [42, 46]) and moderate to high intensity AE (60% of HRmax [45], 60–90% of HRmax [47], 70–75% of HRR [41, 42, 46]). Thus, we can speculate that the reason why moderate to high intensity subgroups mostly conducting combination of AE and RE showed less increase in FMD is not just due to inclusion of RE to AE. Our results could provide opposing views against the previous studies placing more weight on the high intensity of AE in order to improve EF, and ultimately can suggest the possibility of low to moderate training as a new alternative therapeutic strategy for T2D patients.

However, in order to determine whether low to moderate intensity training can be an alternative treatment for T2D patients, further studies are required. Because original studies had a small sample size and this current study evaluated some even smaller subgroups of the original studies. Of 7870 potential studies, only eight studies were included for our meta-analysis by narrowing inclusion criteria, which could increase a risk of both bias and extrapolation of the results. On the other hand, heterogeneity between studies decreased due to the small number of well conducted studies in this field. Thus, one of the main positive aspects of this study was to uncover poor evidence on this field and assume a critical position.

Meanwhile, our results show that there was no significant difference in the effect of training duration on FMD between eight and twelve or more weeks, which indicates that vascular EF in T2D patients could be improved by exercise training for a relatively short period of time. Also, we can speculate that overweight or obese (≥ 30 kg/m^2^) T2D patients need to have different exercise prescription from normal weight patients (< 30 kg/m^2^) because improvement of FMD in subgroups with low BMI levels (< 30 kg/m^2^) was higher compared to in subgroups with high BMI levels (≥ 30 kg/m^2^). Though each 10 kg decrease in body weight was correlated with 1.1% increase in fasting FMD [64], our results suggest that FMD can be increased by exercise training without weight loss. In regard to training frequency, almost all trials performed 3 days per week so that we cannot further analysis.

Although AE may be currently the most effective exercise modality for improving EF, focusing largely on AE cannot be a recommended treatment for most T2D patients who have the risk of CVD. Recent studies reported that low skeletal muscle mass is associated with increase in arterial stiffness [65, 66]. Ohara et al. [67] also reported that thigh muscle cross-sectional area in 1470 older adults significantly and independently correlated with arterial stiffness measured by brachial pulse wave velocity. In particular, T2D patients should take into account that glucose uptake and glycogen storage predominantly occurred in skeletal muscle [68]. Furthermore, considering two facts, (1) those aged 65 and older account for the largest proportion of total T2D patients [69]; (2) muscle weakness starts at age 50 [70] and more aggravates by age 65 [71], it is certainly necessary for T2D patients, especially elderly to improve vascular function and increase muscular strength and mass together in order to not only reduce the risk of CVD but also improve glycemic control, their fundamental problems. Therefore, we believe that combination of AE and RE should be considered as more optimized strategy for most T2D patients rather than unitary exercise modality. Further studies need to be warranted to devise new modality of combined exercise, such as circuit training being consist of AE and RE or low intensity-high repetition RE with short rest times or active recovery to enhance oxygen utilization and muscle protein synthesis at the same time.

There are some limitations in our study. First, in the EX, those who have other intervention, such as ET receptor blockade, meditation, and hypoxic environment were included. Although these supplementary interventions did not make significant effects on FMD, it could cause bias. Second, the methods measuring FMD are somewhat different between research groups, which might influence on the results. Third, only one study [42] was blinded for outcomes, diminishing the quality of studies. Fourth, there is the possibility of the Hawthorne effect influencing the results. However, there is not any control group in included studies which reported significant change in all outcomes although the control groups participated in a study but did not perform exercise training. Moreover, one of the inclusion criteria for eligible studies was training for at least 8 weeks, averagely 12 weeks which may be sufficient to induce certain physiological responses to exercise. Thus, we speculate that the Hawthorne effect might be trivial. Lastly, the number of studies included in our study may not be adequate, which could augment a risk of bias, however significant publication bias was not found in all variables.

We first investigated effects of exercise training on both vascular EF as well as shear rate AUC, baseline diameter, and BMI in T2D patients. The study design is valuable because on the basis of the finding, we can suggest low to moderate intensity training can be an alternative strategy for improving EF. Second, we performed meta-analysis based on studies targeting only T2D patients where EF is assessed by only brachial artery FMD, the major predictor for CVD and atherosclerosis, which importantly provides the validity of the results. Thus, we believe that this study extends our knowledge to provide an optimized therapeutic strategy to reduce the risk of CVD in T2D patients.

## Conclusion

This systemic review and meta-analysis found that exercise training significantly increased brachial artery FMD in T2D patients without adverse events, but there was no change in shear rate AUC, baseline diameter, and BMI. Subgroups with low to moderate intensity increased FMD more than moderate to high intensity subgroups, suggesting that increase in NO production and bioavailability does not solely depend on an increase in shear stress, and the possibility of low to moderate training as a new alternative therapeutic strategy for T2D patients. There is an impressive small number of well conducted studies in this field. Further studies are needed to establish more optimized exercise prescription guideline for T2D patients.

## Abbreviations

AE: aerobic exercise
AUC: area under the curve
BMI: body mass index
CI: confidence interval
CON: control group
CONT: continuous training
CVD: cardiovascular disease
EF: endothelial function
eNOS: endothelial nitric oxide synthase
ES: effect size
ET: endothelin
EX: exercise group
EXP: experimental group
FMD: flow-mediated dilation
GRADE: Grading of Recommendations Assessment, Development and Evaluation
HbA1c: glycated hemoglobin
HRmax: maximum heart rate
HRR: hear rate reserve
INT: interval training
METs: metabolic equivalents
NO: nitric oxide
RE: resistance exercise
SD: standard deviation
T2D: type 2 diabetes
VO_2peak_: peak oxygen consumption
1-RM: one-repetition maximum
